# Electroretinographic Findings in Fragile X, Premutation, and Controls: A Study of Biomarker Correlations †

**DOI:** 10.3390/ijms26146830

**Published:** 2025-07-16

**Authors:** Hasan Hasan, Hazel Maridith Barlahan Biag, Ellery R. Santos, Jamie Leah Randol, Robert Ring, Flora Tassone, Paul J. Hagerman, Randi Jenssen Hagerman

**Affiliations:** 1 Medical Investigation of Neurodevelopmental Disorders (MIND) Institute, University of California Davis, 2825 50th Street, Sacramento, CA 95817, USA; hbbiag@ucdavis.edu (H.M.B.B.); ersantos@ucdavis.edu (E.R.S.); ftassone@ucdavis.edu (F.T.); pjhagerman@ucdavis.edu (P.J.H.); 2 Department of Clinical Neurosciences, Salmaniya Medical Complex, Manama 00973, Bahrain; 3 Department of Pediatrics, School of Medicine, University of California Davis, 4610 X St, Sacramento, CA 95817, USA; 4 Department of Biochemistry and Molecular Medicine, School of Medicine, University of California Davis, 4610 X St, Sacramento, CA 95817, USA; jlrandol@ucdavis.edu; 5 Kaerus Bioscience Ltd., 168 Shoreditch High Street, London E1 6RA, UK; robert.ring@kaerusbio.com

**Keywords:** electroretinography (ERG), fragile X syndrome, biomarkers, *FMR1* premutation, FMRP

## Abstract

The study’s aim was to evaluate electroretinographic (ERG) alterations in Fragile X syndrome (FXS), *FMR1* premutation carriers, and controls, and to explore correlations with peripheral blood FMRP expression levels and behavioral outcomes. ERG recordings were obtained using a handheld device across three stimulus protocols in 43 premutation carriers, 39 individuals with FXS, and 23 controls. Peripheral blood FMRP expression levels were quantified using TR-FRET (Time-Resolved Fluorescence Resonance Energy Transfer). Correlations were assessed with cognitive and behavioral measures including IQ (Intelligence Quotient), ABC_FX_ (Aberrant Behavior Checklist for Fragile X Syndrome), SNAP-IV (Swanson, Nolan, and Pelham Teacher and Parent Rating Scale), SEQ (Sensory Experiences Questionnaire), ADAMS (Anxiety, Depression, and Mood Scale), and the Vineland III Adaptive Behavior Scale standard score. Significant group differences were observed in multiple ERG parameters, particularly in 2 Hz b-wave amplitude (*p* = 0.0081), 2 Hz b-wave time to peak (*p* = 0.0164), 28.3 Hz flash combined amplitude (*p* = 0.0139), 3.4 Hz red/blue flash b-wave amplitude (*p* = 0.0026), and PhNR amplitude (*p* = 0.0026), indicating both outer and inner retinal dysfunction in FXS and premutation groups. Despite high test–retest reliability for ERG (ICC range = 0.71–0.92) and FMRP (ICC = 0.70), no correlation was found between ERG metrics and FMRP or behavioral measures. However, FMRP levels strongly correlated with IQ (ρ = 0.69, *p* < 0.0001) and inversely with behavioral impairment [ABC_FX_ (ρ = −0.47, *p* = 0.0041), SNAP-IV (ρ = −0.48, *p* = 0.0039), SEQ (ρ = −0.43, *p* = 0.0146), and the Vineland III standard score (ρ = 0.56, *p* = 0.0019)]. ERG reveals distinct retinal functional abnormalities in *FMR1*-related conditions but does not correlate with peripheral FMRP expression levels, highlighting the need for multimodal biomarkers integrating radiological, physiological, behavioral, and molecular measures.

## 1. Introduction

Fragile X syndrome (FXS) is the most common inherited form of intellectual disability and a leading monogenic cause of autism spectrum disorder (ASD). It results from a CGG trinucleotide repeat expansion (>200 repeats) in the 5′ untranslated region of the *FMR1* gene, which is typically accompanied by hypermethylation and gene silencing. The consequent absence or severe reduction in fragile X messenger ribonucleoprotein (FMRP)—a critical RNA-binding protein—leads to widespread dysregulation of synaptic function and neuronal excitability [1]. In individuals with *FMR1* CGG repeat and/or methylation mosaicism, and in females with one affected X chromosome, FMRP levels vary according to repeat size, methylation status, and X-inactivation ratios. Those carrying a premutation allele (55–200 CGG repeats) typically exhibit elevated *FMR1* mRNA levels and mildly reduced FMRP expression, particularly in the upper premutation range, due to inefficient translation [2]. In individuals with FXS, there is a documented impairment in the magnocellular (M) pathway, processing motion, and low spatial frequency information [3,4]. In premutation carriers, there is selective impairment of the M pathway, where they exhibit lower contrast sensitivity and higher thresholds for motion-defined stimuli [5,6,7]. The parvocellular (P) pathway that processes high spatial frequency and color information is relatively spared in both groups.

Beyond its role in FXS, FMRP deficiency is also observed in other neurodevelopmental and psychiatric conditions, including idiopathic ASD and schizophrenia, where its reduction correlates with cognitive dysfunction [8,9,10,11]. FMRP is known to regulate the translation of numerous proteins involved in synaptic plasticity, including many associated with ASD [12,13]. Recent studies have demonstrated that FMRP directly modulates large-conductance calcium- and voltage-activated potassium (BK) channels, which play a critical role in regulating neuronal excitability [14]. Loss of FMRP results in BK channelopathy, leading to excessive neuronal firing, impaired synaptic homeostasis, and increased glutamate release—mechanisms that may underlie key symptoms of FXS and related disorders [15,16].

In the past decade, progress has been made in understanding the role of FMRP in both vision and FXS. FMRP is expressed in all the layers of the retina, with notable expression in the Müller cells—the principal glial cells of the retina [17]. These cells are involved in the glutamate-glutamine metabolic cycle, which is crucial for neurotransmitter recycling in the retina. In the absence of FMRP, there is evidence of disruption in neurotransmitter homeostasis, including lower glutamine synthetase expression and activity in the Müller cells, leading to decreased conversion of glutamate to glutamine for neuronal uptake [18]. Moreover, FMRP deficiency in the retina results in the dysregulation of 944 genes, with 409 genes upregulated and 535 genes downregulated [19]. Interestingly, FMRP expression in the retina is modulated by light. On light exposure, photoreceptors are hyperpolarized, decreasing glutamate release by these cells, and subsequently decreasing phosphorylation and degradation of FMRP in the retina [20]. FMRP represses translation of rhodopsin through association with mRNA encoding rhodopsin (ninaE mRNA). Light exposure triggers calcium-dependent dephosphorylation of FMRP, relieving the suppression of rhodopsin translation [21].

Electroretinography (ERG), a non-invasive technique for assessing retinal function, has emerged as a promising translational biomarker for FMRP-related neural dysfunction [22]. A recent report has demonstrated that the absence of FMRP in *Fmr1* knockout mice appears to protect against age-related decline in retinal function. Specifically, these mice did not exhibit the typical age-related reduction in ERG a- and b-wave amplitudes observed in wild-type mice, suggesting that FMRP may play a role in the aging process of the retina by influencing oxidative stress and free radical production [23].

BK channels are widely expressed in the retina, and studies in *Fmr1* knockout mice have revealed ERG b-wave deficits, mirroring visual contrast sensitivity abnormalities observed in patients with FXS [24,25]. These findings have been validated in human studies, where reduced ERG b-wave amplitudes were identified in individuals with FXS compared to neurotypical controls [22]. Furthermore, the sensitivity of ERG abnormalities to BK channel modulation in preclinical models supports its utility as a target-engagement biomarker in therapeutic trials.

Given that FMRP expression levels vary across individuals with full mutation, premutation, and control alleles, ERG may provide a window into central nervous system (CNS) function that correlates with peripheral measures of FMRP. This is particularly relevant because the retina and brain share a common origin from the embryological diencephalon. Preliminary data suggest that FMRP expression levels in peripheral blood mononuclear cells (PBMCs), quantified using techniques such as Time-Resolved Fluorescence Resonance Energy Transfer (TR-FRET), are associated with cognitive and behavioral features in FXS [26]. However, systematic studies investigating the relationship between FMRP levels, ERG abnormalities, and neurobehavioral phenotypes across the full mutation and premutation spectrum remain limited.

In this study, we build on previous work [27] to investigate electroretinographic abnormalities in individuals with FXS, premutation carriers, and neurotypical controls, and to examine their correlation with peripheral FMRP expression levels. By integrating electrophysiological and molecular data, we seek to demonstrate the potential of ERG as a CNS biomarker for FMRP-related dysfunction and explore its utility in characterizing phenotypic variability and guiding therapeutic development.

## 2. Results

A total of 105 participants, including 43 premutation carriers, 39 individuals with FXS, and 23 controls, were recruited into this study. One participant with FXS with an a-wave amplitude more than −1 µV was excluded from analysis. Test-retest reliability was assessed in a subset of participants, specifically two healthy controls and one premutation carrier. Descriptive statistics for participant characteristics and ERG parameters are summarized in Table 1.

### 2.1. Group Differences in ERG Parameters Across Diagnostic Categories

**Figure 1:**
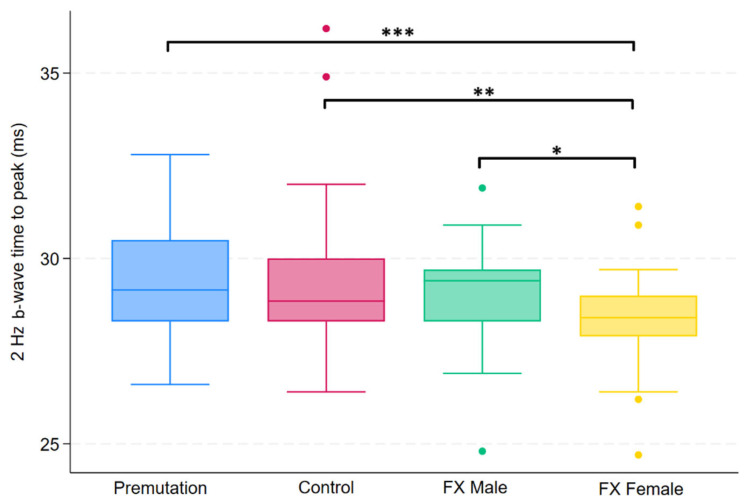
Boxplot illustrating group differences in 2 Hz b-wave time to peak (ms) among premutation carriers, controls, and individuals with Fragile X syndrome (FX males and FX females). Kruskal–Wallis tests identified significant overall differences (*p* = 0.0164). Pairwise comparisons were conducted using Dunn’s post hoc test, with *p*-values adjusted using the Benjamini–Hochberg procedure to control the false discovery rate. Asterisks indicate significant pairwise comparisons: *p* < 0.05 (*), *p* < 0.01 (**), *p* < 0.001 (***).

**Figure 2:**
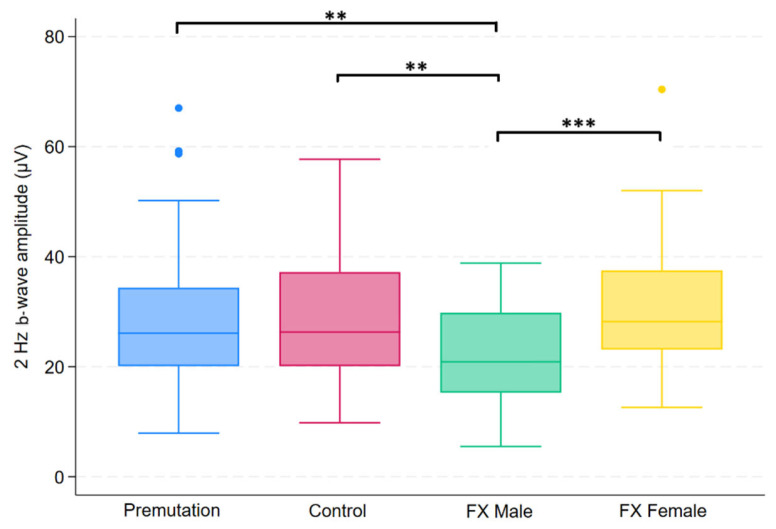
Boxplot illustrating group differences in 2 Hz b-wave amplitude (µV) among premutation carriers, carriers, and individuals with Fragile X syndrome (FX males and FX females). Kruskal–Wallis tests identified significant overall differences (*p* = 0.0081). Pairwise comparisons were conducted using Dunn’s post hoc test, with *p*-values adjusted using the Benjamini–Hochberg procedure to control the false discovery rate. Asterisks indicate significant pairwise comparisons: *p*< 0.01 (**), *p* < 0.001 (***).

**Figure 3:**
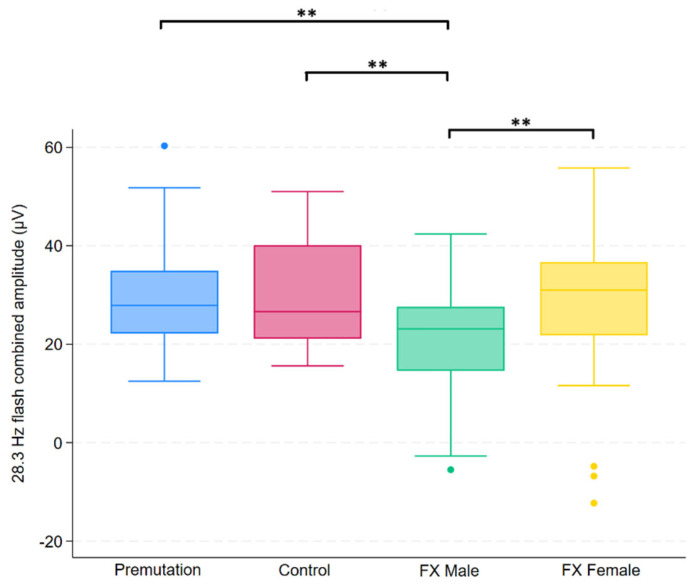
Boxplot illustrating group differences in 28.3 Hz flash combined amplitude (µV) among premutation carriers, controls, and individuals with Fragile X syndrome (FX males and FX females). Kruskal–Wallis tests identified significant overall differences (*p* = 0.0139). Pairwise comparisons were conducted using Dunn’s post hoc test, with *p*-values adjusted using the Benjamini–Hochberg procedure to control the false discovery rate. Asterisks indicate significant pairwise comparisons: *p* < 0.01 (**).

**Figure 4:**
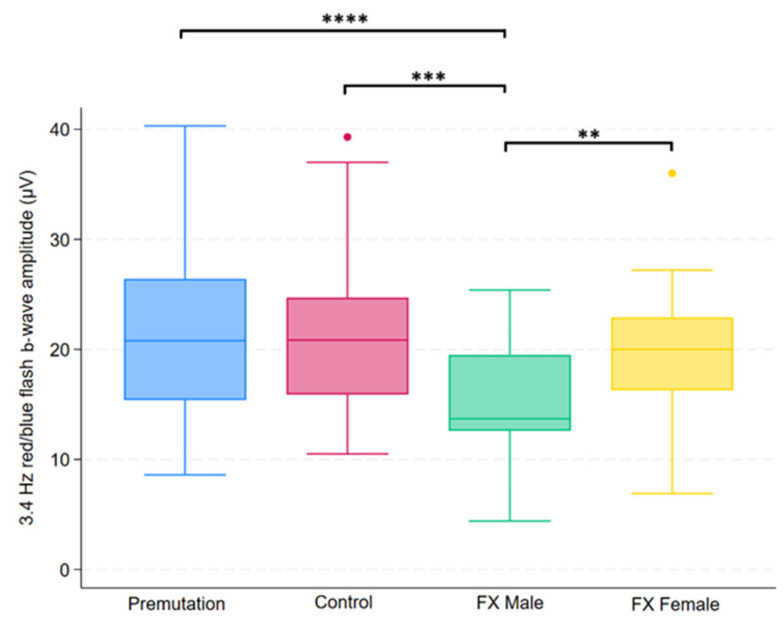
Boxplot illustrating group differences in 3.4 Hz red/blue flash b-wave amplitude (µV) among premutation carriers, carriers, and individuals with Fragile X syndrome (FX males and FX females). Kruskal–Wallis tests identified significant overall differences (*p* = 0.0026). Pairwise comparisons were conducted using Dunn’s post hoc test, with *p*-values adjusted using the Benjamini–Hochberg procedure to control the false discovery rate. Asterisks indicate significant pairwise comparisons: *p* < 0.01 (**), *p* < 0.001 (***), *p* < 0.0001 (****).

**Figure 5:**
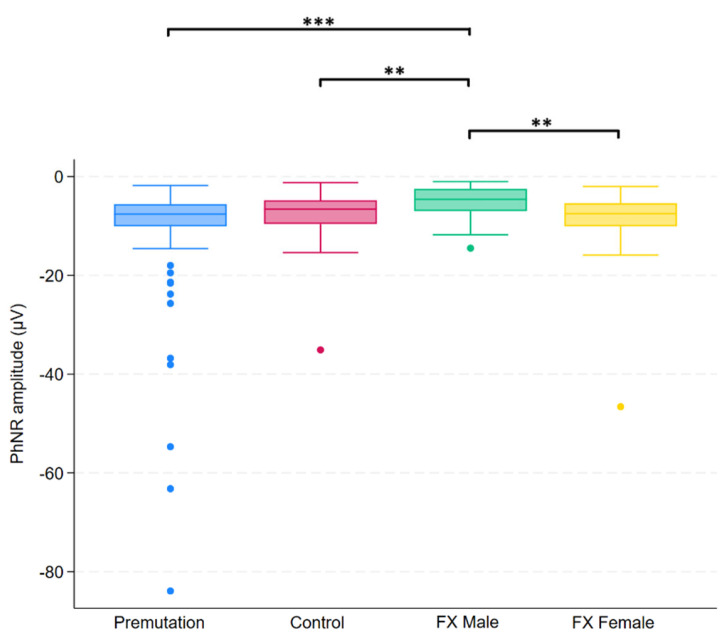
Boxplot illustrating group differences in PhNR amplitude (µV) among premutation carriers, controls, and individuals with Fragile X syndrome (FX males and FX females). Kruskal–Wallis tests identified significant overall differences (*p* = 0.0026). Pairwise comparisons were conducted using Dunn’s post hoc test, with *p*-values adjusted using the Benjamini–Hochberg procedure to control the false discovery rate. Asterisks indicate significant pairwise comparisons: *p* < 0.01 (**), *p* < 0.001 (***).

Group differences in FMRP levels were assessed using the Kruskal–Wallis test across four groups. Of the 14 variables tested, five showed statistically significant differences after applying the Benjamini–Hochberg procedure to control the false discovery rate (FDR = 0.05). These variables were 3.4 Hz red/blue flash B-wave amplitude, 2 Hz B-wave amplitude, 2 Hz B-wave time to peak, 28.3 Hz flash combined amplitude, and PhNR amplitude. Post hoc comparisons for these variables were conducted using Dunn’s test, with group-specific differences summarized in Figure 1, Figure 2, Figure 3, Figure 4 and Figure 5.

### 2.2. Test-Retest Reliability of FMRP and ERG Parameters

**Figure 6:**
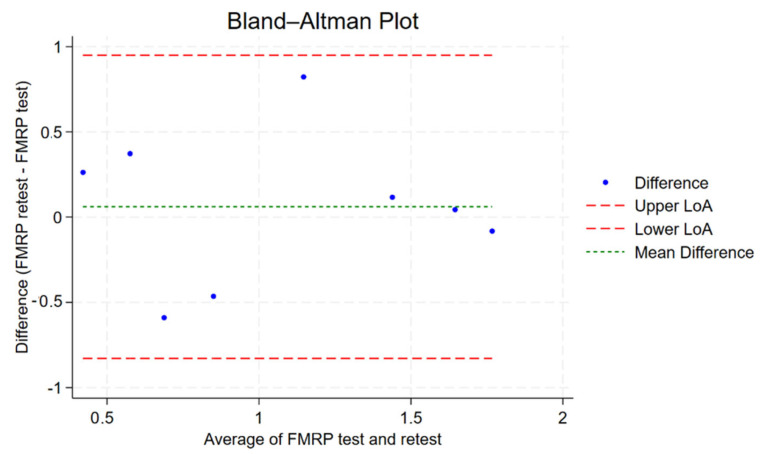
Bland–Altman plot showing the agreement between FMRP test and retest measurements. Each dot represents the difference between retest and test values plotted against their average. The green dashed line indicates the mean difference (bias), while the red dashed lines represent the upper and lower limits of agreement (LoA) (mean ± 1.96 × SD). Most observations fall within these limits, suggesting good test–retest reliability without significant bias or heteroscedasticity.

A two-way random-effects ICC model assessing absolute agreement between the FMRP test and retest yielded an individual ICC of 0.702 and an average ICC of 0.825, indicating good to very good reliability. Average ICC denotes the reliability of the average of the FMRP test and retest in reflecting the true score. The ICC was statistically significant (*p* = 0.014), suggesting that the observed agreement is unlikely due to chance. The Bland–Altman plot of absolute differences in FMRP measurements shows minimal mean bias (mean difference ~−0.14), indicating no systematic over- or underestimation between test and retest (see Figure 6). The limits of agreement (approximately −1.04 to +0.77) indicate that most values fall within acceptable bounds, supporting good test–retest reliability. [Interpretation of Intraclass Correlation: ICC < 0.40 = poor reproducibility, 0.40 ≤ ICC < 0.75 = fair to good reproducibility, ICC ≥ 0.75 = excellent reproducibility] [28].

Test-retest reliability of ERG parameters in the first healthy control was good on the same-day testing protocol (first and second sessions in the morning separated by ten minutes, with two sessions in the evening as well separated by ten minutes) (ICC = 0.71, *p* = 0.008), with excellent repeatability for average ICC reaching 0.83. When a session from two months prior was included, individual-level reliability decreased to moderate levels (ICC = 0.49, *p* = 0.005), while the average ICC remained high (0.83). These findings suggest that the ERG shows strong same-day repeatability and retains stable performance over longer intervals when results are averaged.

In the second healthy control, ERG parameters demonstrated good short-term repeatability within the morning session, with an individual ICC of 0.77 (*p* = 0.037) and an average ICC of 0.87, indicating excellent reliability when averaged across measurements. When compared to a session conducted two months earlier, individual-level reliability dropped to poor (ICC = 0.29, *p* = 0.222), reflecting increased biological variability over time. Evening session data were excluded from the final analysis due to missing values and unstable ICC estimates, which compromised model validity.

Test-retest reliability was also tested in a premutation carrier for a morning session thirty minutes apart, showing excellent short-term repeatability, with an individual ICC of 0.92 (*p* = 0.040) and an average ICC of 0.96, indicating highly consistent measurements when averaged.

### 2.3. Group Differences in FMRP Levels

**Figure 7:**
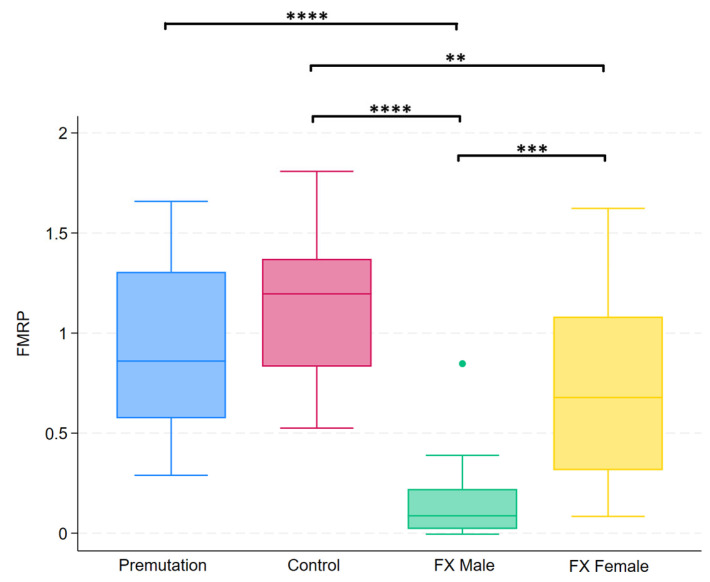
Boxplots illustrating group differences in FMRP levels among premutation carriers, controls, and individuals with Fragile X syndrome (males and females). Kruskal–Wallis tests identified significant overall differences between groups. Pairwise comparisons were conducted using Dunn’s post hoc test. Asterisks indicate significant pairwise comparisons: *p* < 0.01 (**), *p* < 0.001 (***), *p* < 0.0001 (****).

Group differences in FMRP levels were assessed using the Kruskal–Wallis test, which revealed a statistically significant difference among the four groups (*p* < 0.0001). Post hoc analysis using Dunn’s test revealed specific group differences, which are summarized in Figure 7.

### 2.4. Correlation of ERG with FMRP Levels, IQ, SEQ, SNAP-IV, ABC, ADAMS, and Vineland III Scores

We averaged ERG amplitudes across the left and right eyes to generate a single representative value per participant. This approach was supported by strong inter-eye correlations across most parameters, with Spearman ρ values exceeding 0.75 (*p* < 0.001) in all categories except for the 2 Hz and 3.4 Hz b-wave amplitudes in the FXS group (ρ = 0.54 and 0.56, respectively), which still demonstrated statistically significant moderate correlations. Averaging was thus considered a valid and robust method for minimizing measurement noise and capturing a unified electrophysiological signal reflective of overall retinal function for correlation.

To examine the stability and internal consistency of retinal responses across different ERG protocols, we correlated b-wave amplitudes obtained from three testing conditions. Strong positive correlations were observed between the 2 Hz b-wave average amplitude and the 28.3 Hz flash average amplitude (ρ = 0.7378, *p* < 0.0001), between the 2 Hz b-wave average amplitude and the 3.4 Hz red/blue flash b-wave amplitude (ρ = 0.7774, *p* < 0.0001), and between the 28.3 Hz flash average amplitude and the 3.4 Hz red/blue flash b-wave amplitude (ρ = 0.7552, *p* < 0.0001).

None of the ERG parameters demonstrated statistically significant correlations with FMRP expression levels, IQ scores, ABC_FX_ Composite scores, SNAP-IV, SEQ, ADAMS, or Vineland III scores (see Table 2).

### 2.5. Correlation Between Molecular and Cognitive/Behavioral Measures

**Figure 8:**
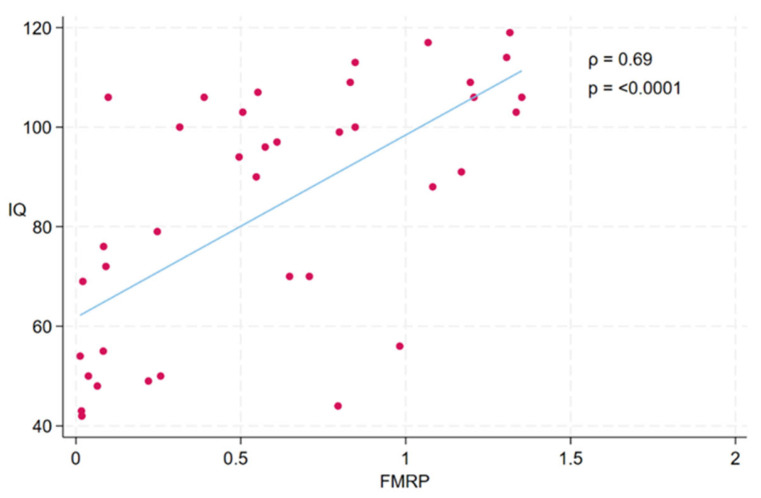
Scatterplot illustrating the association between FMRP levels and IQ scores across participants. Each dot represents an individual participant. A significant positive correlation was observed between FMRP and IQ (Spearman’s ρ = 0.69, *p* < 0.0001), indicating that higher levels of FMRP were associated with higher cognitive function as measured by IQ. The fitted line represents the linear trend between the two variables.

A statistically significant strong positive correlation between FMRP levels and IQ scores was observed in the study participants (ρ = 0.69, *p* < 0.0001) (see Figure 8). Participants with higher FMRP levels tend to have higher IQ scores.

Additionally, FMRP negatively correlated with the ABCFX composite score (ρ = −0.47, *p* = 0.0041), SNAP-IV (ρ = −0.48, *p* = 0.0039), SEQ (ρ = −0.43, *p* = 0.0146), and Vineland III standard score (ρ = 0.56, *p* = 0.0019). FMRP did not correlate with ADAMS total score (ρ = −0.32, *p* = 0.12).

Behavioral scales were strongly intercorrelated, with ABC_FX_ and SNAP-IV (ρ = 0.86, *p* < 0.0001), ABC_FX_ and SEQ (ρ = 0.82, *p* < 0.0001), SEQ and SNAP-IV (ρ = 0.85, *p* < 0.0001), ADAMS and SNAP-IV (ρ = 0.90, *p* < 0.0001), ADAMS and SEQ (ρ = 0.82, *p* < 0.0001), ADAMS and ABC_FX_ (ρ = 0.84, *p* < 0.0001) demonstrating high convergence. The Benjamini–Yekutieli correction was applied to control the false discovery rate, and all correlations remained statistically significant following adjustment. No statistically significant correlations were observed between the Vineland III standard score and SNAP-IV (ρ = −0.25, *p* = 0.26), SEQ (ρ = 0.03, *p* = 0.89), ABC_FX_ (ρ = −0.09, *p* = 0.69), and ADAMS total score (ρ = −0.41, *p* = 0.12)

### 2.6. Association Between Age and PhNR Response

Based on prior studies showing age-related effects on retinal responses [29,30], we assessed whether PhNR amplitude or time to peak correlated with age to repurpose it as a longitudinal biomarker in FXTAS. There was no significant correlation between age and either PhNR amplitude (ρ = 0.03, *p* = 0.76) or PhNR time to peak (ρ = 0.07, *p* = 0.45). Upon stratifying the sample by diagnostic group, no statistically significant associations were observed between age and PhNR parameters. In the premutation group, no significant correlations were observed between age and PhNR amplitude (ρ = 0.12, *p* = 0.42) or time to peak (ρ = −0.01, *p* = 0.91). Among FX males, there was no significant association between age and PhNR amplitude (ρ = 0.14, *p* = 0.59) or time to peak (ρ = −0.03, *p* = 0.91). In FX females, no significant correlations were observed between age and PhNR amplitude (ρ = 0.55, *p* = 0.051) or PhNR time to peak (ρ = −0.14, *p* = 0.64). Similarly, in neurotypical controls, neither PhNR amplitude (ρ = 0.32, *p* = 0.14) nor time to peak (ρ = −0.06, *p* = 0.77) demonstrated significant age-related correlations.

## 3. Discussion

The primary outcome of the study was to study the correlation between neurophysiological measures (b-wave amplitudes across three testing protocols) and molecular/cognitive measures. The lack of correlation can be explained by tissue-specific expression and regulation of FMRP in the retina. There is differential expression of FMRP in the body, with high levels in the brain compared to the peripheral lymphocytes [31]. Peripheral blood FMRP levels may not accurately represent retinal FMRP, especially considering the retina’s unique metabolic and neurochemical profile. This is bolstered by findings that in FXS, the retina has a unique transcriptomic profile with few common genes to the CNS [19]. It is important to consider the natural fluctuations in both electrophysiological measures and protein expression when interpreting the results of this study. The absence of a clear ERG-FMRP correlation does not negate the potential of ERG as a non-invasive biomarker of neurophysiological function. It, however, emphasizes the importance of integrating ERG within a multimodal biomarker framework that includes neuroimaging, behavioral measures, and molecular assays to capture the full complexity of FXS.

To our knowledge, this is the first study to underscore the heterogeneity of electrophysiological phenotypes in premutation carriers, FX males, FX females, and healthy controls. Significant group differences were observed in several ERG parameters, notably in 2 Hz b-wave amplitude and time to peak, 28.3 Hz combined b-wave amplitude, 3.4 Hz red/blue flash b-wave amplitude, and PhNR amplitude. These findings suggest both outer and inner retinal dysfunction, particularly in individuals with full mutation FXS, and to a lesser extent in premutation carriers. The reduction in b-wave amplitude in FX males is consistent with previously reported findings. Previous work by Perche et al. reported reduced b-wave and flicker amplitudes in males with FXS (n = 20) compared to healthy controls (n = 20) [22]. Similarly, a recent conference abstract described significantly lower 2 Hz flash and 28.3 Hz flicker b-wave amplitudes in the FXS group (n = 21) relative to controls (n = 16) [32].

Unlike the consistent ERG abnormalities observed across studies in FXS, findings in other neurodevelopmental conditions have been more variable. In one study evaluating ERG as a biomarker in ADHD (attention deficit hyperactivity disorder), no significant differences were noted between ADHD participants (n = 26) and controls (n = 25) overall, though sex-specific effects emerged [33]. Males with ADHD showed prolonged cone a-wave latency, and females with ADHD showed decreased cone a- and b-wave amplitudes along with prolonged cone b-wave latency. Another study investigating ERG in high-functioning ASD (n = 32), failed to detect significant differences in ERG a-wave, b-wave, or PhNR parameters compared to controls (n = 31). This discrepancy with other studies investigating the use of ERG as a biomarker with ASD may be due to differences in sample size, age, diagnostic heterogeneity, testing conditions (dark-adapted or light-adapted ERG), and methodological discrepancies (whether b-wave amplitudes were averaged across eyes or the highest b-wave amplitude was used). In contrast, other studies reported attenuated a- and b-waves in ASD along with no changes in PhNR amplitude suggesting normal ganglion cell function in ASD [34,35,36]. A recent study investigated ERG responses in individuals with ADHD, ASD, and neurotypical controls using a light-adapted protocol [37]. This study reported statistically significant elevations in b-wave amplitude and PhNR responses in ADHD compared to both control and ASD groups. In addition, the ADHD group exhibited a faster b-wave time to peak, ASD showed a delayed response, and controls lay in between. These findings point toward differential imbalances in glutamatergic and GABAergic neurotransmission in ASD and ADHD which have likely contributed to the distinct ERG profiles observed.

To assess whether the lack of correlation between ERG b-wave amplitudes and peripheral blood FMRP levels was due to measurement variability, we evaluated test-retest reliability for both measures. The results indicated good to excellent reliability for FMRP and ERG parameters, particularly when averaged across repeated measurements. This suggests that measurement noise or instability is unlikely to explain the absence of association. Notably, while short-term repeatability was high, individual-level reliability decreased over longer intervals in some cases, consistent with expected biological fluctuation. Prior literature similarly reports generally good consistency for ERG parameters, with ICCs ranging from 0.48 to 0.92, supporting the robustness of ERG as a physiological biomarker [38]. Additionally, intra-visit reliability for ERG amplitudes using the RETeval system has been reported to be high (ICC = 0.79), reinforcing its consistency within a single session [39]. Discrepancies in measurement acquisition time have also been shown to have no significant effect on retest outcomes in light-adapted RETeval ERG, suggesting that strict time-of-day standardization may not be necessary for follow-up assessments [40]. Prior studies have shown that FMRP levels are highly reproducible across repeated measurements using techniques such as quantitative sandwich ELISA and Luminex-based immunoassays, with values remaining stable over months [41,42] Consistent with this, our test–retest analysis using a TR-FRET–based assay demonstrated good to very good reliability, further supporting the stability of FMRP as a peripheral molecular marker. This level of reproducibility reduces the likelihood that random measurement variability accounts for the absence of correlation between FMRP and ERG.

We examined whether PhNR amplitude declined with age in all participants and in FMR1 premutation carriers to assess its potential as a longitudinal biomarker in FXTAS. No significant association was found. Notably, our result aligns with previous studies reporting no correlation between age and PhNR amplitude in healthy adults [43] and showing only weak, non-significant trends after correction for multiple comparisons [44]. In contrast, studies that have identified age-associated changes in PhNR, such as increased time to peak or amplitude decline, have largely been conducted in disease-specific cohorts like primary open-angle glaucoma [29] or in aging mouse models [30]. This limits their generalizability to neurodevelopmental or premutation carrier populations. While age correlations are a common cross-sectional approach, they reflect inter-individual differences and may not capture within-subject decline, which is more relevant for tracking progression. Thus, our negative finding does not rule out PhNR’s longitudinal utility but underscores the need for repeated-measures studies.

We aimed to explore whether the lack of correlation between ERG and FMRP or behavioral measures reflected a true dissociation rather than measurement noise or limited variability. To address this, we examined correlations among ERG b-wave measures across sessions and stimuli, and separately assessed correlations between molecular and cognitive/behavioral markers. The strong correlations of b-wave amplitudes confirm that ERG signals are stable and reproducible across testing conditions, supporting their biological validity. Similarly, robust associations between FMRP expression levels and behavioral severity (IQ, ABC_FX_, SNAP-IV, SEQ), as well as high intercorrelations among the behavioral scales themselves, further affirm the internal consistency of these domains. Positive correlations among the behavioral scales also likely reflect their partial overlap in measuring externalizing behaviors, sensory dysregulation, and regulatory challenges common in FXS. Together, these findings indicate that the absence of correlation between ERG and FMRP or behavioral scores is unlikely due to methodological limitations and more likely reflects a true dissociation between ERG and FMRP/behavioral scales.

We observed a strong positive correlation between FMRP levels and IQ, consistent with previous studies showing that higher FMRP expression is associated with better cognitive function [41,45,46,47]. Consistent with prior literature, we observed significant negative correlations between FMRP expression levels and ABC_FX_ composite score, indicating greater behavioral impairment with lower protein expression. A previous study similarly found that higher *FMR1* mRNA levels were associated with greater irritability and poorer quality of life in FX males with incomplete silencing, which reinforces the link between molecular dysregulation and behavioral phenotype severity in fragile X-associated conditions [48]. While our findings revealed significant correlations between FMRP expression levels and behavioral measures composite scores (ABC_FX_, SNAP-IV), previous studies, such as [49], reported no significant associations with subscale scores [50]. This discrepancy may be due to methodological differences, including the use of composite versus subscale scores and a wider range of FMRP expression in our mixed diagnostic sample. Aggregation of behavioral symptoms into composite scores may provide greater sensitivity to molecular associations by capturing a broader behavioral phenotype. However, on the other hand, each symptom does not contribute equally to the composite score and some subscales with more items may disproportionately drive the total. In addition, ceiling effects may limit the ability to differentiate individuals with extreme scores, potentially reducing sensitivity to detect associations particularly at the upper extremes of the behavior spectrum.

### Limitations and Future Directions

While the majority of participants (older than ten years old) tolerated the ERG procedure well, one male with full mutation FXS was unable to complete it. Nonetheless, this relatively low attrition supports the feasibility of handheld ERG using skin electrodes without requiring corneal electrodes or sedation. However, tactile hypersensitivity may be triggered by skin adhesive electrodes, especially in pediatric participants where adult-sized stickers are extended onto the temple and hairline [51]. Future development of pediatric-specific, sensory-friendly electrodes along with desensitization strategies could improve tolerability. The use of a Troland-based protocol, which adjusts luminance in real-time based on pupil diameter, may be disrupted by erratic eye movements common in neurodevelopmental populations leading to termination of the test. In such cases, a fixed-intensity candela protocol could serve as a more consistent alternative by delivering standard retinal stimulation independent of pupil tracking [51]. Strict procedural standardization regarding electrode placement is required as a minor deviation of a few millimeters beneath the lower eyelid can affect waveform morphology and time-to-peak measurements. This would reduce operator-dependent variability and ensure comparability across studies and sites. We did not account for refractive errors, which can reduce ERG amplitudes—especially in cases of high myopia or hyperopia—and may confound interpretation of retinal function [52,53]. Future studies should consider assessing visual acuity and refractive status, such as hyperopia commonly seen in FXS [54,55], when interpreting ERG results.

As this was an exploratory study, formal power calculations were not performed. However, the effect sizes observed here—specific to the statistical tests used—can serve as preliminary estimates to guide future sample size planning. Given that effect sizes depend on the analysis method and sample characteristics, and are subject to variability, future studies should use sensitivity analyses and account for uncertainty in effect size estimates when planning sample size [56]. Longitudinal studies assessing ERG parameters particularly PhNR in individuals with FXTAS (Fragile X-associated tremor/ataxia syndrome) may help determine whether ERG can serve as a multimodal (along with imaging and metabolic) biomarker for neurodegeneration over time. In addition, future studies with larger samples should explore whether ERG parameters correlate with CGG repeat size in methylation mosaic individuals, and whether sex and methylation % interact in predicting ERG measures. Such analyses would require adequately powered mosaicism samples to enable robust regression modeling. ERG can be explored in individuals with other rare genetic BK channelopathies, including mutations in *KCNMA1*, *KCNMB4*, *CRBN*, and the *FMR1* R138Q variant (mutation at the site of FMRP binding to BK channel), to assess whether similar retinal dysfunction patterns to FXS are observed. Importantly, future research should explore how FMRP deficits and RNA toxicity mechanistically impact ERG outcomes. The effect of age as a covariate or moderator in ERG outcomes and FMRP-related phenotypes requires larger sample sizes to allow for age-based stratified analyses.

OCT (Optical Coherence Tomography) provides high-resolution, cross-sectional images of the retinal layers. OCT and ERG have been correlated in various conditions, particularly retinal disorders [57,58,59,60]. It is increasingly being utilized in the study of neurodegenerative and neurodevelopmental conditions. OCT studies have shown that adults with ASD exhibit reduced macular and outer nuclear layer thickness, which inversely correlates with the severity of symptoms as measured by the Social Responsiveness Scale 2 (SRS-2) [61]. Additionally, OCT has been proposed for its potential in early diagnosis of inflammatory changes in the central nervous system in children with ASD [62]. While OCT shows promise in the context of ASD, its application in FXS and FXTAS remains unexplored and warrants further investigation. OCT provides structural information, while ERG provides physiological function—together, they add to a growing repertoire of multimodal biomarkers that can offer complementary insights into retinal and CNS involvement in fragile X-associated conditions.

## 4. Materials and Methods

### 4.1. Study Design

This was a cross-sectional, observational case-control study with correlational analysis designed to examine differences in electrophysiological measures of CNS function using ERG among individuals with the *FMR1* premutation, Fragile X syndrome (FXS) males and females, and healthy controls.

Correlation analyses were conducted between ERG measures and peripheral blood levels of FMRP. Additionally, associations were explored between ERG measures and clinical assessments relevant to BK channel dysfunction, including

- Behavioral and Sensory Evaluations:
  ○ Vineland Adaptive Behavior Scales, Third Edition (VABS-III) (Pearson Assessments, San Antonio, TX, USA)
  ○ Stanford-Binet Intelligence Scales, Fifth Edition (SB5) (PRO-ED Inc., Austin, TX, USA),
- Questionnaire-Based Measures:
  ○ Aberrant Behavior Checklist for Fragile X syndrome (ABC_FX_)
  ○ Anxiety, Depression, and Mood Scale (ADAMS) (NovoPsych, Melbourne, Australia)
  ○ Sensory Experiences Questionnaire 3.0 (SEQ-3.0)
  ○ Swanson, Nolan, and Pelham Teacher and Parent Rating Scale (SNAP-IV)

Participants were recruited over a 3-year period (2022–2025), including both newly diagnosed and previously known patients, through the UC Davis MIND Institute research and clinical programs. During the same visit, the comprehensive assessment protocol included a blood draw for determining CGG repeat allele size, methylation status, and FMRP expression levels with an on-site phlebotomist; ERG evaluation; physical and neurological examination; behavioral/sensory evaluation (SB-5 and ADOS-2 if not conducted in the recent past with the VABS-III); and questionnaire evaluations (ABC_FX_, SEQ, SNAP-IV, and ADAMS). The primary outcome was the correlation between b-wave amplitude (measured from the ERG) and FMRP blood expression levels. Other secondary outcomes include group differences between premutation carriers, full mutation, and controls in ERG measures. Additionally, test-retest reliability of FMRP and ERG measures was also assessed amongst a few participants at multiple time periods during the day. This involved testing using the same adhesive skin electrode on the ERG, taken ten minutes apart, as well as a third measurement after six hours using a separate pair of electrodes. FMRP levels were assessed at separate visits.

All participants (or their legal guardians) provided written informed consent prior to study participation.

### 4.2. Participants

The study included participants with either the *FMR1* premutation or full mutation.

Eligibility for the study was determined based on several criteria. Genetic confirmation was required, with molecular genetic testing verifying the presence of either the full *FMR1* mutation (>200 CGG repeats) or *FMR1* premutation (55–200 CGG repeats). The control group underwent genetic testing to rule out the presence of a premutation.

Participants included males and non-pregnant, non-lactating females aged 3 to 75 years (inclusive). All participants (or their legal guardians) provided written informed consent prior to study participation. For minors or individuals lacking legal capacity, a parent or caretaker was required to provide consent and agree to participation. Additionally, premutation participants and caretakers for those with FXS needed the ability to read, write, and speak English fluently to complete study-related materials and demonstrate reliable attendance at research visits.

Participants were excluded if they had any eye disease affecting the retina that could interfere with ERG measurements. Those with life-threatening medical conditions or systemic illnesses that could compromise their health, safety, or ability to complete the study were also excluded. Finally, individuals younger than 3 years or older than 75 years were not eligible for participation.

### 4.3. ERG Protocol

ERG recordings were obtained using the RETeval^®^ device (LKC Technologies, Inc. Gaithersburg, MD, USA). This is a handheld portable device designed for performing ERG in pediatric individuals at any age, without requiring mydriasis, corneal electrodes, or sedation. It has been validated in several studies generating results similar to the classical ERG device [22,51,63,64,65]. Testing takes 10–15 min to complete on both eyes. The RETeval^®^ device has its device-specific reference data and was well-tolerated by patients with FXS [22]. During testing, participants were seated comfortably while the periorbital skin was prepped with Nuprep gel to reduce electrode impedance. The individual’s ID and birthdate were entered into the device, and the sensor strip barcode was scanned. A self-adhesive electrode was applied 1–4 mm beneath the lower eyelid, aligned medially with the pupil for accurate positioning. The electrode was connected, and a soft eye cup was placed over the eye to be tested, while the opposite eye was occluded by the participant’s hand. The device’s built-in photometer automatically locates the pupil, and once alignment was confirmed via an on-screen green circle, the start button activated. The test began upon user initiation. The process was then repeated for the opposite eye, with the participant switching hands. If pupil detection failed due to blinking or misalignment, electrode adjustments were made and the test was reinitiated. All tests were performed under consistent ambient room lighting (LA—light-adapted condition). Upon completion, the results were displayed, automatically saved as PDFs, and securely uploaded to ShareFile via Kaerus Bioscience Ltd. (London, UK) The RETeval^®^ device generates 14 ERG parameters, all of which were included in the analysis.

For this study, the peak amplitudes (μV) and time to peak in milliseconds (ms) of ERG a- and b-waves for the ISCEV (International Society for Clinical Electrophysiology of Vision) standard were assessed. The first test involved a white flash at an intensity of 85 Troland-seconds of retinal illuminance delivered at a flash frequency of 2 flashes per second on a background light intensity of 850 Td. Rod photoreceptors were fully suppressed, allowing an isolated response from cone photoreceptors. The first test evaluated cone-mediated function under light-adapted conditions [66,67,68].

The second test involved white flicker at an intensity of 85 Tds at a frequency of 28.3 Hz. At high flash frequencies (>25 Hz), rods cannot respond due to their slower temporal resolution (temporal resolution—ability to detect changes in response to visual stimuli over time). Cones can process fast flickering light, ensuring an isolating cone function without interference from rods. This was also performed on a steady 850 Td background light throughout the test. Unlike single-flash ERG, which captures a-wave (photoreceptor) and b-wave (bipolar/Müller cells), flicker ERG evaluates cone-driven temporal processing [69,70,71]. In flicker ERG, the rapid, repetitive stimuli do not allow sufficient time for full a-wave recovery before the next response occurs, causing overlapping signals and preventing clear resolution of the a-wave. Because the retina does not return to baseline between flashes, the photoreceptor response is effectively summed over multiple cycles, making it difficult to isolate direct cone-origin (a-wave) activity. Instead, b-wave measurements here reflect cone-driven post-synaptic responses, primarily from bipolar and Müller cells. In essence, flicker ERG evaluates how well bipolar and Müller cells process cone signals, rather than directly assessing cone photoreceptor function. The primary contributors to the flicker ERG are the bipolar cells, with Müller cells playing a minor role in influencing the amplitude and shape of the b-wave [72].

The third test evaluated outer retinal function (a-wave by the photoreceptor rods and cones, b-wave by the bipolar and Müller cells) and inner retinal function (Photopic Negative Response or PhNR), particularly the ganglion cell layer [73,74,75,76]. The test used a red (long-wavelength) flash of 38 Tds with the background light being blue (short-wavelength) at 380 trolands. Red light primarily stimulates L-cones (long-wavelength-sensitive cones). At this intensity, a measurable response was generated from the cones without saturating them. Blue background light suppresses rods and M-cones (medium wavelength cones) while keeping L-cones responsive. This ensures that the response measured comes from the L-cones and the downstream pathways involving the retinal ganglion cells reducing noise from other photoreceptor populations in the measurement of PhNR [77,78]. The time to peak, amplitude, and W-ratio were measured for the Photopic Negative Response. The W-ratio helps assess the relative strength of the PhNR compared to the preceding b-wave, providing insight into retinal ganglion cell function. A lower W-ratio (closer to 0) suggests significant ganglion cell dysfunction, manifesting as PhNR suppression. A deeper PhNR relative to the b-wave would reflect preserved ganglion cell function—W-ratio closer to 1.0 or higher [79].

(1) $$W-ratio=\frac{b-P_{min}}{b-a}$$

where:

- *a* = a-wave peak (photoreceptor response)
- *b* = b-wave peak (bipolar and Müller cell response)
- *P_min_* = minimum of the PhNR wave (ganglion cell response)

**Figure 9:**
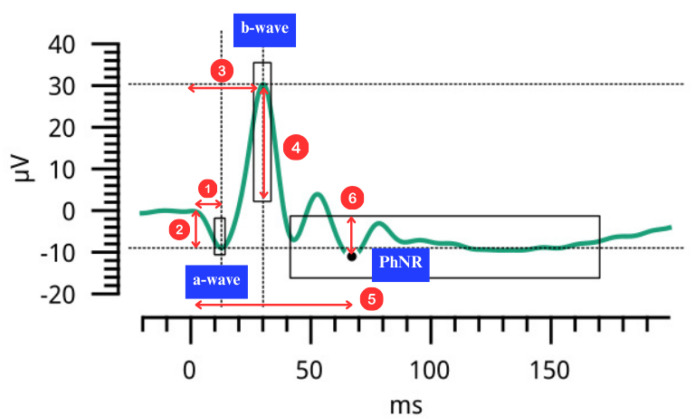
Electroretinography (ERG) waveform depicting the negative a-wave, positive b-wave and the Photopic Negative Response (PhNR). Numbered arrows depict (1) a-wave time to peak (2) a-wave amplitude (3) b-wave time to peak (4) b-wave amplitude (5) PhNR time to peak (6) PhNR amplitude.

ERG waveforms are depicted in Figure 9. ERG assessment outcomes included time to peak (in milliseconds, ms) and amplitude (in microvolts, µV) for different testing conditions:

- Single Flash (First Portion of the Test): a-wave and b-wave time to peak and amplitude were measured for both eyes.
- Flicker Stimulus (Second Portion of the Test): b-wave time to peak and amplitude were recorded in µV.
- Red Flash on Blue Background (Third Portion of the Test): Outcomes included a-wave, b-wave, and PhNR (Photopic Negative Response) time to peak and amplitudes, as well as the W-ratio.

### 4.4. TR-FRET Assay for FMRP Levels

The TR-FRET method used to quantify FMRP was as described in [49], with the following modification: in the Aishworiya et al. paper, samples were normalized to the mean of all samples with normal alleles, whereas, for the current work, samples were normalized to the mean of a single (fiducial) PBMC (peripheral blood mononuclear cell) sample, collected and banked from a single blood draw and run on each plate.

### 4.5. FMR1 CGG Repeat Allele Size and Methylation Status

CGG repeat allele size and methylation status were assessed using a combination of Southern blot analysis and PCR amplification. For Southern blot analysis, 10 μg of genomic DNA isolated from peripheral blood leukocytes was digested with EcoRI and NruI, run on an agarose gel, transferred to a nylon membrane, and hybridized with the FMR1-specific dig-labeled StB12.3. PCR analysis was carried out using FMR1-specific primers (AmplideX PCR/CE, Asuragen, Inc., Austin, TX, USA) and the amplicons, visualized by capillary electrophoresis, were analyzed using Gene Mapper software^TM^ (version 4.0). Details of these methods have been previously reported [80,81].

### 4.6. Statistical Analysis

Statistical analyses were conducted using Stata (version 18, StataCorp LLC, College Station, TX, USA). The Shapiro-Wilk test and Levene’s test of homogeneity of variances revealed that the data violated assumptions of normality and homogeneity of variances; therefore, non-parametric tests were chosen. Kruskal–Wallis test was used to differentiate ERG parameters between premutation carriers, males with FXS, females with FXS, and healthy controls. Dunn’s post hoc test was used as a follow-up to determine pairwise group comparisons. Test-retest repeatability of FMRP levels in 9 subjects was assessed using the intraclass correlation coefficient ICC(2,1), based on a two-way random-effects model, as measurement occasions were considered random samples from a broader set of possible time points. In contrast, repeatability of ERG parameters was assessed in 3 subjects (2 healthy controls and 1 premutation carrier) using ICC(3,1), based on a two-way mixed-effects model, since the measurements were taken at fixed time intervals. Bland–Altman plots were used to visualize the agreement between measurements and identify any systematic biases. The appropriateness of this method was confirmed by evaluating the absence of proportional bias and heteroscedasticity, ensuring that key model assumptions were met [82]. Spearman correlation coefficients were used to assess correlations between specific ERG outcomes (average b-wave amplitude for the three parts of the test) and FMRP levels, IQ, SEQ, SNAP-IV, and ABC_FX_ scores, as well as the correlation between FMRP levels and IQ for all participants. *p*-Value ≤ 0.05 was considered to be statistically significant. Interpretation of ICC values followed conventional thresholds: <0.50 (poor), 0.50–0.75 (moderate), 0.75–0.90 (good), and >0.90 (excellent) reliability. Participants with a-wave amplitude greater than −1 μV were excluded from analysis. Benjamini–Hochberg correction was applied to control the false discovery rate for multiple group comparisons across ERG parameters. This method was deemed appropriate given the independence of ERG test protocols and group assignments (premutation carriers, FXS males, FXS females, and controls). Benjamini–Yekutieli procedure was applied to control the false discovery rate under dependency across multiple Spearman correlation tests between behavioral scales and biological/ERG variables. As this was an exploratory study, formal sample size calculations were not conducted. Multiple imputation was not used for missing values.

## 5. Conclusions

In this exploratory study, we observed distinct patterns of ERG alterations in individuals with FXS, premutation carriers, and controls. This was observed especially in the b-wave and PhNR amplitudes, reflecting both outer and inner retinal dysfunction. While ERG measures did not correlate with peripheral FMRP levels or behavioral scores, FMRP levels were strongly associated with cognitive and behavioral profiles. These findings reinforce the potential of ERG as a physiological biomarker in fragile X-associated conditions. The findings also underscore the importance of integrating multiple modalities–including ERG, imaging, molecular assays, and behavioral evaluations for a more comprehensive understanding of *FMR1*-related neurobiology. Longitudinal studies are warranted to further characterize the progression of these retinal changes.

## Figures and Tables

**Table 1:** Descriptive statistics of participants in the study. All variables are presented as median (IQR) due to non-normal distributions in at least one study group, as assessed by the Shapiro-Wilk test. Although the 3.4 Hz red/blue flash a-wave time to peak was normally distributed across groups, a consistent reporting format was used for ease of comparison. Notably, 28.3 Hz flash data are presented as combined amplitude (both eyes) and best amplitude (highest of left/right eye). W-ratio and FMRP are unitless.

Variable	Premutation	Fragile X	Control
Age (years) Median (IQR)	53 (24)	16 (20)	35 (19)
2 Hz a-wave time to peak (ms)	11.9 (1.3)	11.6 (1.5)	11.9 (1.5)
2 Hz a-wave amplitude (μV)	−7.6 (3.9)	−6.7 (3.6)	−6.5 (4.5)
2 Hz b-wave time to peak (ms)	29.1 (2.2)	28.6 (1.4)	28.8 (1.7)
2 Hz b-wave amplitude (μV)	26.1 (14.2)	24.3 (14.4)	26.3 (17.1)
28.3 Hz flash time to peak (ms)	25.5 (2.0)	25.3 (1.3)	25.3 (1.0)
28.3 Hz flash combined amplitude (μV)	27.9 (12.7)	27.2 (14.6)	26.6 (19)
28.3 Hz flash best amplitude (μV)	28.9 (14.5)	28.8 (13.3)	32.9 (19.5)
3.4 Hz red/blue flash a-wave time to peak (ms)	12.0 (1.0)	12.1 (1.3)	12 (1.6)
3.4 Hz red/blue flash a-wave amplitude (μV)	−5.0 (2.7)	−4.2 (2.0)	−4.5 (2.7)
3.4 Hz red/blue flash b-wave time to peak (ms)	28.7 (2.5)	28.3 (2.6)	28.1 (2.7)
3.4 Hz red/blue flash b-wave amplitude (μV)	20.8 (11)	17.3 (8.7)	20.8 (8.8)
PhNR time to peak (ms)	78 (50)	82.5 (65)	73 (12)
PhNR amplitude (μV)	−7.6 (4.5)	−6.5 (4.4)	−6.6 (4.8)
W-ratio	1.13 (0.22)	1.1 (0.17)	1.11 (0.12)
FMRP	0.86 (0.73)	0.22 (0.58)	1.19 (0.53)

**Table 2:** Spearman correlation coefficients between ERG parameters and clinical variables (FMRP—fragile X messenger ribonucleoprotein; IQ—Intelligence Quotient; ABC_FX_—Aberrant Behavior Checklist for Fragile X syndrome; SEQ—Sensory Experiences Questionnaire; SNAP-IV—Swanson, Nolan, and Pelham Questionnaire-IV; ADAMS—Anxiety, Depression, and Mood Scale).

ERG Parameter	Correlated Measure	Spearman’s ρ	*p*-Value
2 Hz b-wave flicker average amplitude (μV)	FMRP	0.06	0.59
	IQ	−0.15	0.34
	ABC_FX_ composite	0.19	0.25
	SEQ	0.18	0.31
	SNAP-IV	0.15	0.36
	ADAMS	−0.12	0.56
	Vineland III	0.27	0.19
28.3 Hz b-wave flash average amplitude (μV)	FMRP	0.05	0.62
	IQ	0.04	0.78
	ABC_FX_ composite	−0.0002	0.99
	SEQ	−0.09	0.63
	SNAP-IV	0.05	0.77
	ADAMS	−0.19	0.36
	Vineland III	0.43	0.06
3.4 Hz red/blue flash b-wave amplitude (μV)	FMRP	0.15	0.16
	IQ	0.05	0.74
	ABC_FX_ composite	0.04	0.78
	SEQ	−0.23	0.21
	SNAP-IV	−0.01	0.92
	ADAMS	−0.36	0.07
	Vineland III	0.12	0.59

## Data Availability

The datasets presented in this article are not readily available due to ongoing studies involving participant data. Requests to access the datasets should be directed to Ellery R. Santos, ersantos@ucdavis.edu.

## Abbreviations

The following abbreviations are used in this manuscript:

ERG	Electroretinography
FXS	Fragile X syndrome
FMRP	Fragile X messenger ribonucleoprotein
TR-FRET	Time-Resolved Fluorescence Resonance Energy Transfer
IQ	Intelligence Quotient
ABC_FX_	Aberrant Behavior Checklist for Fragile X Syndrome
SNAP-IV	Swanson, Nolan, and Pelham Teacher and Parent Rating scale
SEQ	Sensory Experiences Questionnaire
ADAMS	Anxiety, Depression, and Mood Scale
ASD	Autism Spectrum Disorder
PBMCs	Peripheral blood mononuclear cells
CNS	Central Nervous System
VABS-III	Vineland Adaptive Behavior Scales
SB5	Stanford-Binet Intelligence Scales
LA	Light adapted
ISCEV	International Society for Clinical Electrophysiology of Vision
PhNR	Photopic Negative Response
ICC	Intraclass Correlation Coefficient
IQR	Interquartile Range
FDR	False Discovery Rate
ADHD	Attention Deficit Hyperactivity Disorder
FXTAS	Fragile X Tremor Ataxia Syndrome
OCT	Optical Coherence Tomography
SRS-2	Social Responsiveness Scale
ADOS-2	Autism Diagnostic Observation Schedule, Second Edition
BK channel	Large-conductance calcium- and voltage-activated potassium channels
ELISA	Enzyme-Linked Immunosorbent Assay
S.D.	Standard Deviation
LoA	Limits of Agreement
